# Focused Real-Time Ultrasonography for Nephrologists

**DOI:** 10.1155/2017/3756857

**Published:** 2017-02-02

**Authors:** Matthew J. Kaptein, Elaine M. Kaptein

**Affiliations:** Division of Nephrology, Department of Medicine, University of Southern California, Los Angeles, CA, USA

## Abstract

We propose that renal consults are enhanced by incorporating a nephrology-focused ultrasound protocol including ultrasound evaluation of cardiac contractility, the presence or absence of pericardial effusion, inferior vena cava size and collapsibility to guide volume management, bladder volume to assess for obstruction or retention, and kidney size and structure to potentially gauge chronicity of renal disease or identify other structural abnormalities. The benefits of immediate and ongoing assessment of cardiac function and intravascular volume status (prerenal), possible urinary obstruction or retention (postrenal), and potential etiologies of acute kidney injury or chronic kidney disease far outweigh the limitations of bedside ultrasonography performed by nephrologists. The alternative is reliance on formal ultrasonography, which creates a disconnect between those who order, perform, and interpret studies, creates delays between when clinical questions are asked and answered, and may increase expense. Ultrasound-enhanced physical examination provides immediate information about our patients, which frequently alters our assessments and management plans.

## 1. Background

Real-time ultrasonography has become an invaluable extension of the physical examination. The keys to learning to incorporate ultrasonography into the bedside evaluation are hands-on practice, supervision to ensure accuracy of the technique, and focusing on the basic examination relevant to the patient evaluation.

The RUSH (Rapid Ultrasound in SHock) and FAST (Focused Assessment with Sonography for Trauma) protocols are routinely used for the immediate assessment and management of unstable patients [1–3]. Pertinent to nephrology, the RUSH exam focuses on the heart to assess for contractility, pericardial effusion, or tamponade, and the inferior vena cava (IVC) maximum diameter and collapsibility to estimate intravascular volume and to guide volume management [4]. The ultrasound views used in the RUSH and FAST exams to assess for free fluid collections in the abdomen can be used to assess the bladder and kidneys.

The RUSH protocol has been shown to aid in a more rapid and reliable diagnosis of shock etiology (Table 1) [3–5]. In a prospective study, the overall sensitivity of the RUSH exam for diagnosing shock was 88% and specificity was 96%, compared to the final diagnosis [5]. Ultrasound findings influenced management [4, 6–8] and were  useful in guiding volume administration or restriction and pressor therapy, which resulted in improved 28-day patient survival, a reduction in stage 3 acute kidney injury, and more days alive and free of renal support [9].

Nephrologists routinely use bedside ultrasonography to localize the kidney for percutaneous renal biopsy and to guide dialysis catheter placement [10]. In patients with end-stage renal disease receiving chronic hemodialysis therapy, ultrasound to determine IVC diameter and collapsibility may be a helpful tool for estimation of intravascular volume status or “dry weight” [11–16].

We have developed our approach by incorporating previously described point-of-care ultrasound evaluations and personal experience.

## 2. Limited Cardiac and Inferior Vena Cava Ultrasound

### 2.1. Procedures and Technical Steps

**Figure 1:**
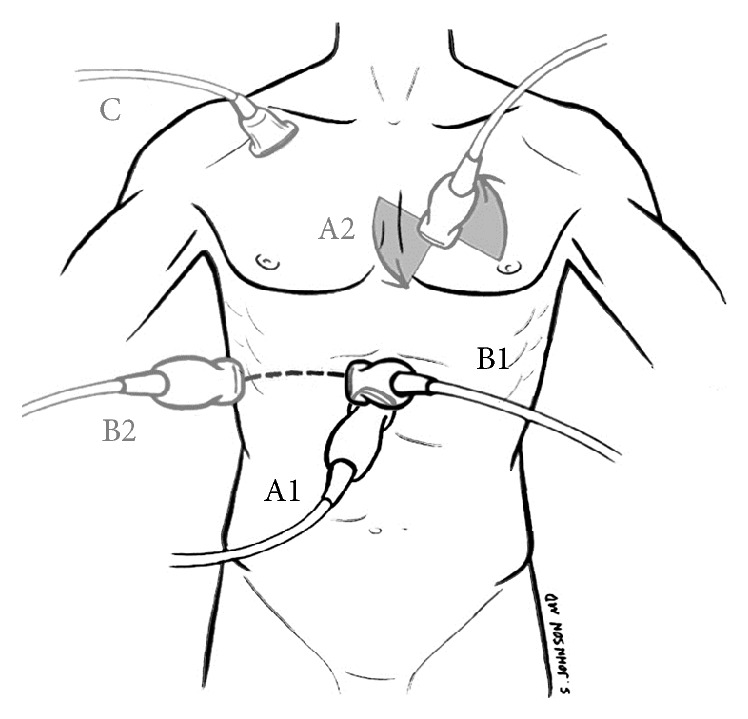
Prerenal assessment: cardiac contractility and intravascular volume. A1 = subcostal cardiac view (curvilinear or phased-array probe), A2 = parasternal long- and short-axis views (phased-array), B1 = IVC long-axis view (curvilinear or phased-array), B2 = IVC long-axis from midaxillary line view (curvilinear or phased-array), and C = subclavian vein view (high-frequency linear probe) [25]. Adapted from Perera et al. with the authors' permission [4].

**Figure 2:**
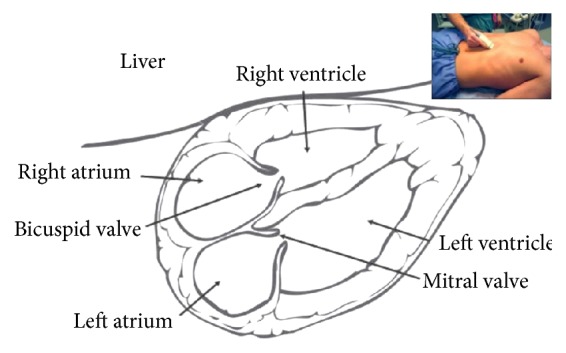
Subcostal cardiac landmarks. Subcostal view is a good window for locating the right atrium prior to the IVC, is useful for qualitative assessment of cardiac contractility, and is sensitive for detecting pericardial effusion or tamponade (frequently unsuspected). As in all transthoracic cardiac views, the right ventricle is closest to the ultrasound probe (see Figure 1, probe position A1). Reproduced from http://www.sonoguide.com/FAST.html 10/08/2016.

We begin by visualizing the heart using the subcostal approach in most patients, with a curvilinear (abdominal) or phased-array (cardiac) probe (Figures 1 and 2). The subcostal view of the heart is good for a visual estimate of ventricular contractility and shows the presence or absence of a circumferential pericardial effusion or tamponade as indicated by diastolic right ventricular collapse. With a cardiac probe, the parasternal long-axis view can be used to differentiate pericardial effusion from pleural effusion, and cardiac output can be visually estimated by ventricular contractility and mitral valve excursion [17]. Formal echocardiography may be required to confirm any suspicious finding or to further evaluate cardiac function.

**Figure 3:**
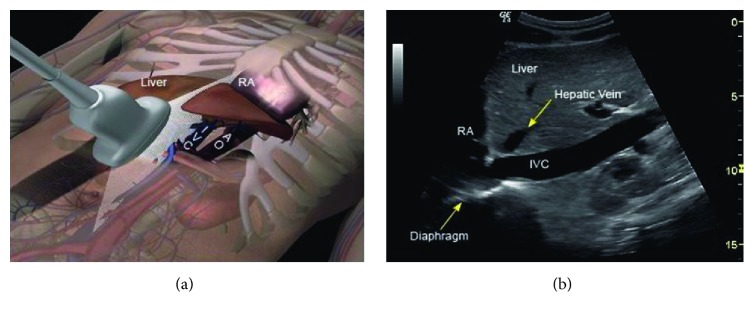
Subcostal inferior vena cava landmarks. (a) Position of ultrasound probe for visualization of the inferior vena cava (IVC) (see Figure 1, probe position B1). The IVC is located to the right of midline and aorta (AO). (b) Corresponding ultrasound image of the IVC. The IVC is typically measured 2 cm from the right atrium (RA) or just distal to the hepatic vein. The hepatic vein junction to IVC and the IVC junction to right atrium are confirmatory landmarks. Reproduced with permission from Killu et al. [26].

After the heart is assessed in the subcostal window, the probe is rotated vertically and moved 1 to 2 cm to the patient's right, while maintaining visualization of the right atrium, to view the IVC in its long-axis (Figures 1 and 3). We recommend intentionally viewing the aorta to the left of the IVC in every patient to be sure that it is not mistaken for the IVC and visualizing the junction of the IVC with the right atrium [18] (Figure 3). There are very few instances in which the IVC can be seen but not the aorta.

The IVC diameters are recorded over several respiratory cycles with spontaneous respiration or mechanical ventilation [3]. As many critically ill patients cannot perform voluntary maneuvers such as sniff or valsalva, which can accentuate IVC collapse [18, 19], for consistency we do not routinely use these maneuvers. Since most of our hospitalized patients are semirecumbent with the head of the bed elevated to 30 degrees and IVC maximum diameter and collapsibility index have not been shown to be statistically different between supine and 45 degree semiupright positions [20], we avoid time-consuming repositioning and perform our ultrasound exam in whatever supine or semirecumbent position the patient is at the time of evaluation.

In instances of extreme hypovolemia, the IVC may be completely collapsed throughout all or most of the respiratory cycle, making it difficult to definitively visualize. To increase the IVC diameter and improve visualization, the patient's knees may be raised off the bed, or the legs may be raised by tilting the entire bed back 45 degrees so that the head of the bed is level (passive leg raising) [21]. A totally collapsed IVC should occur in conjunction with a totally collapsed hepatic vein.

Care should be taken to maintain adequate visualization of the IVC throughout the respiratory cycle because the relationship of the probe to the IVC may be displaced during deep respiration or abdominal breathing [18, 22] (Table 2). This displacement may be misinterpreted as IVC collapse [18]. In such patients, we recommend sliding the ultrasound probe to the patient's right and looking between the lower anterior ribs, moving towards the midaxillary line [23, 24] (Figure 1). The maximum IVC diameter may be underestimated in the long-axis due to off-center placement of the ultrasound probe (cylinder tangent effect) [18] (Table 2).

The maximum and minimum diameters of the IVC are determined visually and are typically measured 2 cm from the right atrium or just distal to the hepatic vein [18, 28] (Figure 3). Most authors have normalized the difference between the maximum and minimum IVC diameter by dividing by the end-expiratory diameter. The end-expiratory diameter is the maximum diameter in spontaneously breathing patients and the minimum diameter in ventilated patients [29]. IVC maximum diameter (IVCmax) minus minimum diameter (IVCmin) divided by maximum diameter has been called collapsibility index (CI) and may be expressed as a percentage [30–34]. CI = (IVCmax − IVCmin)/IVCmax *∗* 100%. IVC maximum diameter minus minimum diameter divided by minimum diameter has been called distensibility index (DI) and may be expressed as a percentage [29]. DI = (IVCmax − IVCmin)/IVCmin *∗* 100%. Distensibility index and collapsibility index can be interconverted using the following two equations: DI = CI/(100% − CI)*∗*100%. CI = DI/(100% + DI)*∗*100%.

For the sake of consistency and convenience, we calculate collapsibility index for all of our patients, both ventilated and nonventilated. The clinical utility of CI versus DI in spontaneously breathing or ventilated patients has not been directly compared, as far as we know. However the variable DI has a denominator that can approach zero in some patients, which can be cumbersome. There is good reproducibility among physicians with experience applying appropriate technique, with 4–9% variation in measures of collapsibility or distensibility [41, 51].

### 2.2. Clinical Utility of Cardiac and IVC Ultrasound

Using the RUSH protocol, one can evaluate cardiac contractility, pericardial effusion or signs of tamponade, and IVC diameters with respiration/ventilation to differentiate types of shock (Table 1) [3–5] or to evaluate potential causes of hypotension during hemodialysis or ultrafiltration. The heart may be dilated with poor contractility, hypertrophic with good contractility, hyperdynamic, or relatively normal. The etiology of an enlarged heart on physical examination or chest X-ray can be immediately differentiated using bedside ultrasound.

The major clinical value of IVC ultrasound findings is that they frequently eliminate one possibility, of either overt intravascular hypervolemia or hypovolemia, in a given patient. In many hospitalized patients the IVC is either “FAT” (IVCmax >2.1 cm, minimal collapsibility) making intravascular volume depletion unlikely or “flat” (IVCmax ≤ 2.1 cm, >50% collapsibility) making intravascular volume overload unlikely [28]. These IVC ultrasound findings may influence the prediction of whether a patient would benefit from administration of volume, diuretics or ultrafiltration, or neither.

Repeated evaluations of the maximum diameter and collapsibility of the IVC with volume administration or removal can guide ongoing volume management to optimize therapy [3], which in turn may improve morbidity and mortality [52]. With our conventions, we generally aim for IVC collapsibility index in the range of 20% to 50%, acknowledging that there are many potential biases to interpretation and overriding clinical considerations, including acute respiratory distress syndrome or desire to extubate, which may require volume removal, and preload dependent conditions that may require volume loading (Table 2).

### 2.3. Comparison of Techniques to Assess Intravascular Volume and Response to Volume Administration or Removal

The clinical determination of intravascular volume and prediction of response to a volume intervention may be more difficult in hospitalized or critically ill patients since they are frequently not in steady state and may have mismatch between intravascular volume and blood pressure or between intravascular and extravascular volume (Table 3).

#### 2.3.1. Clinical Symptoms and Signs

Medical history, physical findings, and laboratory tests have limited sensitivity and specificity to assess intravascular volume or volume responsiveness [41, 53–55].

#### 2.3.2. Daily Weights, “Net Fluid Inputs and Outputs”

Calculating total volume of “fluids” administered and lost from the body over time does not account for insensible losses, for third space shifts, for differing effects of blood, crystalloids, colloids, or water administration on intravascular volume, or for the varying sodium concentrations of body fluid losses [56, 57]. In patients with acute decompensated heart failure, net “fluid” balance and weight loss have been shown to be poorly correlated and unreliable [58] and may not reflect intravascular volume status.

#### 2.3.3. Chest Radiography and Lung Ultrasound

Physical examination and chest X-ray are of limited utility to evaluate for pericardial effusion, cardiac dysfunction, and intravascular volume. Chest radiography has low sensitivity for volume overload with up to 20% of patients with heart failure having negative findings during their initial evaluation [59] and low specificity given that there is a wide differential for pulmonary infiltrates on chest X-ray. Lung ultrasound may provide additional useful information to differentiate the causes of acute respiratory failure and to guide volume therapy [60–62]. Integrated cardiopulmonary sonography may result in more rapid and better informed clinical decision making, shorten the time to diagnosis of pulmonary edema, and decrease the risk of excessive volume resuscitation [63].

#### 2.3.4. Comparison of “Dynamic” to “Static” Parameters to Predict Responsiveness to Volume Administration or Removal

*Mean values* for central venous pressure (CVP), right atrial pressure (RAP), pulmonary artery occlusion pressure (PAOP), maximum IVC diameter, stroke volume, or cardiac output are “static” parameters and generally have low sensitivity and specificity to assess volume responsiveness [21, 42, 64] (Table 4). The majority of unstable patients no longer have CVP or Swan-Ganz catheters since they have not been shown to improve mortality [9, 21, 42, 64, 65]. Assessment of relative blood volume by online monitoring of hematocrit is a “static” parameter which has little if any value in avoiding intradialytic hypotension during ultrafiltration in hospitalized or critically ill patients with acute kidney injury [66–69].

“Dynamic” parameters, which take into account the respiratory/ventilatory variation of RAP, IVC diameter, stroke volume, systolic blood pressure, or pulse pressure, are better predictors of volume responsiveness [21, 42, 64] (Table 4). All are technologically refined versions of jugular venous waveform (CVP max/min, RAP max/min, and IVC collapsibility) or “pulsus paradoxus” (stroke volume variation, pulse pressure variation). The respiratory/ventilatory variations in these parameters are greater in volume responsive than volume nonresponsive patients.

### 2.4. Comparison of Dynamic Parameters to the “Gold Standard”: An Increase in Cardiac Output

The only purpose of a volume challenge is to increase stroke volume or cardiac index by at least 10–15%, which has been considered a “gold standard” for assessing response to volume administration [41, 53]. Only 50% of hemodynamically unstable critically ill patients respond to volume expansion with a significant increase in stroke volume or cardiac output [41, 53, 64, 70]. There is a need for techniques to differentiate patients who will benefit from volume expansion, from those who are nonresponders and may benefit from inotropic or vasopressor support but not volume therapy [42] or those who may benefit from volume removal using diuretics or ultrafiltration [56].

“Dynamic” parameters such as pulse pressure variation (PPV) and stroke volume variation (SVV) are highly predictive of volume responsiveness, assessed as an increase in cardiac index of at least 10%, under very limited circumstances including mechanical ventilation with tidal volume >8 mL/kg, excluding spontaneous breathing or cardiac arrhythmias [48, 49] (Table 4). PPV and SVV have been less reliable when implemented in automated systems such as FloTrac™/Vigileo™ (Edwards Life Science LLC, Irvine, CA, USA) [47] and have not been validated in hypervolemia. Bioreactance is a noninvasive assessment of SVV, cardiac output, and other variables based on analysis of relative phase shifts of an oscillating current when the current traverses the thoracic cavity, which predicts volume responsiveness in a heterogeneous group of patients [47, 50] (Table 4).

RAP changes over the respiratory cycle predict volume responsiveness as assessed by an increase in cardiac output [44] (Table 4). IVC diameter changes in a similar manner to RAP throughout the respiratory cycle as long as no obstruction or restriction of the vena cava is present. IVC collapsibility is predictive of volume responsiveness [29, 42, 71, 72] or intravascular volume overload [73, 74].

IVC collapsibility performs comparably to other “dynamic” predictors of volume responsiveness such as PPV and SVV when assessed by an increase in cardiac index of at least 10% in ventilated patients (Table 4). Though the ability of IVC parameters to predict volume removal is not well established, preliminary unpublished observational data suggest that the degree of IVC collapsibility correlates inversely with the likelihood of successful ultrafiltration [45]. Volume removal by ultrafiltration has been shown to increase cardiac output in patients with refractory congestive heart failure [75] and to improve ejection fraction in volume overloaded patients with end-stage renal disease [16].

Ultrasound of the heart and IVC are readily performed at the bedside at the time of patient evaluation. The technique is noninvasive and reproducible and facilitates initial evaluation and ongoing assessment of shock and of cardiomegaly and an estimate of intravascular volume, which may help to guide decisions for volume resuscitation or removal by ultrafiltration or diuretics. Other “dynamic” techniques are frequently not available on a day-to-day basis at the time of patient evaluation for volume management.

### 2.5. Limitations of Cardiac and IVC Ultrasound

Bedside cardiac ultrasound is limited by definition and may require formal echocardiography to verify findings. Limitations to IVC ultrasound can be categorized as factors which affect the IVC diameter/collapsibility or its clinical interpretation [42, 76] and those which limit optimal visualization [77]. The former can be addressed by a systematic understanding of the direction of potential biases and interpretation of results in clinical context for a specific patient (Table 2).

### 2.6. Factors That Affect IVC Diameter or Collapsibility

Overestimation of intravascular volume may occur in conditions that impede flow to the right heart, including valvular abnormalities, pulmonary hypertension, heart failure [18], or poor ventilatory excursions [22, 40] (Table 2). In such circumstances, if the IVC is “flat,” intravascular hypovolemia is likely present and volume resuscitation may be indicated. Underestimation of intravascular volume may occur with intra-abdominal hypertension [36]; therefore a large IVC in this circumstance likely indicates intravascular hypervolemia.

Interpretation of vena cava physiology may be hindered by conditions that restrict the physiologic variability of the IVC such as venous thrombosis, masses causing external compression, or large extracorporeal membrane oxygenation (ECMO) catheters [18, 76]. In patients after liver transplant the central venous anatomy is significantly altered, and there are several possible surgical approaches. This has yet to be systematically investigated. Interpretation of the physiologic characteristics of the IVC should be done in context of the patient's clinical scenario and adjunctive data [18].

#### 2.6.1. Factors That Limit Visualization

Adequate visualization may be compromised by morbid obesity, abdominal pain or distention, bowel gas, postoperative surgical dressings, an open chest or abdomen, subcutaneous emphysema, or talcum powder on the skin. Limitation of optimal subcostal visualization may be addressed by expanding the repertoire of alternative ultrasound windows. In such difficult cases, the IVC may be visualized in the right midaxillary or anterior axillary line [24], and points in-between have been used [23] (Figure 1). One study indicates that subclavian vein collapsibility index using a linear probe correlates well with IVC collapsibility (*r*^2^ = 0.61, *r* = 0.78), providing an additional potential option in patients where IVC measurements are not readily obtainable or are technically limited [25] (Figure 1).

## 3. Urinary Bladder Ultrasound

### 3.1. Procedure and Technical Steps

**Figure 4:**
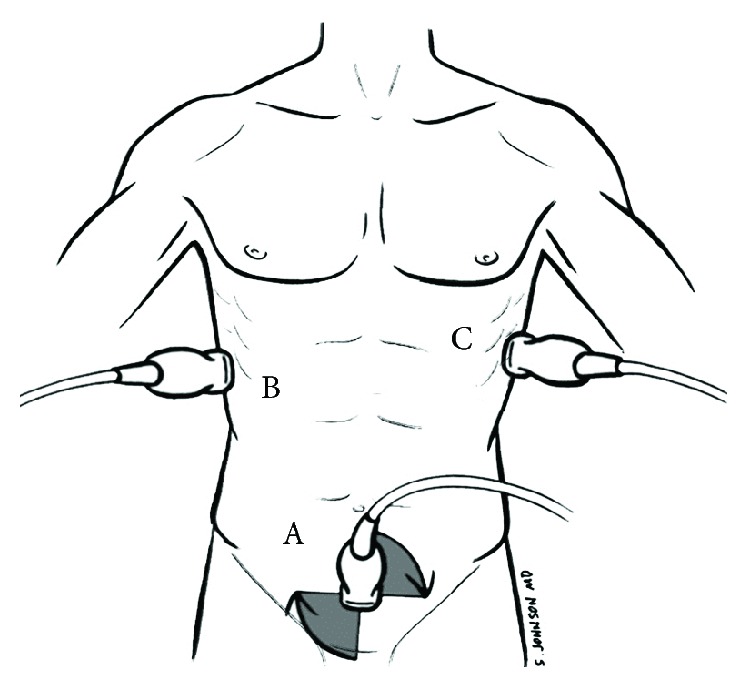
Postrenal/renal assessment: bladder and kidneys. Either a curvilinear or phased-array probe can be used to assess the bladder and kidneys. A = suprapubic view for bladder volume and Foley bulb position, B = RUQ hepatorenal view, and C = LUQ splenorenal view. Adapted from Perera et al. with the authors' permission [4].

**Figure 5:**
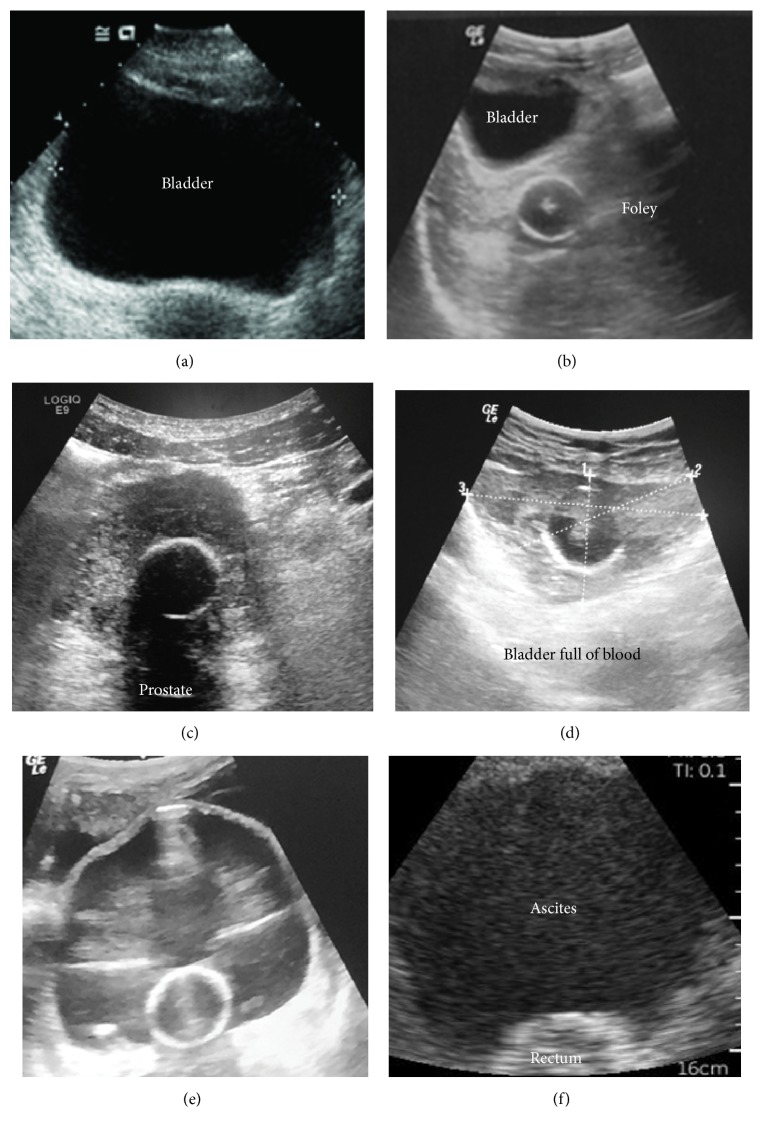
Postrenal assessment: bladder. (a) To calculate bladder volume, the maximum anterior-posterior bladder diameter is measured on an axis perpendicular to that of the longitudinal measurements. Volume (mL) = length (cm) × width (cm) × height (cm) × (0.52 to 0.57) for an ellipsoid (see Figure 4, probe position A). (b) shows a Foley bulb deployed in the pelvis of a patient with anuric renal failure. (c) shows a Foley bulb inflated in the prostate. (d) shows a Foley catheter positioned in a bladder filled with coagulated blood of an anuric patient. (e) Bladder is distended around the Foley bulb due to catheter obstruction. (f) Patient with ascites. Suprapubic view is sensitive for detecting pelvic fluid. It may be difficult to differentiate bladder fluid from ascites with ultrasound.

To assess bladder volume, the abdominal (or cardiac) probe is placed above the symphysis pubis (under the pannus with obesity) and directed caudally towards the prostate or cervix to visualize and measure the maximum transverse and longitudinal bladder diameters (Figure 4). The maximum anterior-posterior bladder diameter is measured on an axis perpendicular to that of the longitudinal measurements. Volume (mL) = length (cm) × width (cm) × height (cm) × (0.52 to 0.57) for an ellipsoid [10, 78, 79]. Many ultrasound machines do this calculation automatically after the three bladder dimensions are defined. We recommend locating the inflated Foley bulb in all catheterized patients (Figure 5). If an indwelling catheter is in the bladder and patent, the Foley bulb should be visible with minimal amounts of urine present in the bladder [79].

### 3.2. Clinical Utility

Urinary retention/obstruction is frequently asymptomatic in patients with acute or chronic renal insufficiency or end-stage renal disease (ESRD) with residual renal function and may predispose to urinary tract infections or contribute to impaired renal function [79]. We propose that bedside ultrasound evaluation of bladder volume be part of the physical exam in all hospitalized patients with acute kidney injury (AKI), chronic kidney disease (CKD), ESRD, or urinary tract infection, and in those who are at risk for urinary retention/obstruction due to urethral stricture, prostatic hypertrophy, neurogenic/atonic bladder, or uterine prolapse. Bladder ultrasound may detect urinary retention/obstruction that is not clinically suspected or confirm the diagnosis when suspected.

Physical exam findings of a suprapubic mass or dullness to percussion may only be detected with bladder volumes greater than 1 liter and may have other etiologies. Even patients with a subjectively “normal” amount of urine production may have significant postvoid residual volume due to partial bladder outlet obstruction or neurogenic bladder.

Assessing bladder volume is particularly critical in the anuric or oliguric patient. Significant residual bladder volume with oliguria or anuria may indicate blockage of the catheter (due to blood clots, debris, or kinked tubing) if the Foley bulb is within the bladder or incorrect placement if the Foley bulb is inflated elsewhere [79] (Figure 5). In an oliguric or anuric patient, a small bladder volume indicates that the indwelling catheter can be removed and ultrasound bladder volumes measured at regular intervals thereafter. Removing unnecessary Foley catheters should decrease urinary tract infections in hospitalized patients [79].

Although estimating a postvoid residual bladder volume is ideal, an estimated bladder volume <100 mL obviates the need for further testing, regardless of the time of the last void. A volume greater than 100 to 150 mL should be rechecked after voiding and, if persistently elevated, confirmed by catheterization [78, 79]. Unnecessary urethral catheterizations can be avoided in patients with small bladder volumes, which should reduce the risk of catheter-related urinary tract infections and urethral trauma [79]. Formal ultrasonography to assess bladder volume after voiding may not be routinely requested if urinary retention/obstruction is not clinically suspected and therefore may be undetected and untreated.

### 3.3. Comparison to the “Gold Standard”: Urethral Catheter Insertion

Bedside bladder ultrasound is an easy to perform, noninvasive method to measure urinary bladder volume without the risk of urethral trauma or urinary tract infection [79]. Mean deviation between ultrasound calculated and voided volume is <10% [78, 79]. A formal ultrasound of the bladder after voiding can be obtained, or the residual volume can be verified using a urethral catheter, as indicated. Large bladder volumes may be detected by ultrasound in patients who are difficult to catheterize.

### 3.4. Limitations

Bladder visualization by ultrasound may be difficult due to abdominal obesity, tissue edema, subcutaneous air, large volume ascites, prior surgery, and suprapubic scarring and after trauma to the anterior abdomen/pelvis in the area of the symphysis pubis [79]. Pelvic fluid such as ascites can be mistaken for urine in the bladder by ultrasonography and can be differentiated with bladder catheterization (Figure 5).

## 4. Limited Renal Ultrasound

### 4.1. Procedure and Technical Steps

**Figure 6:**
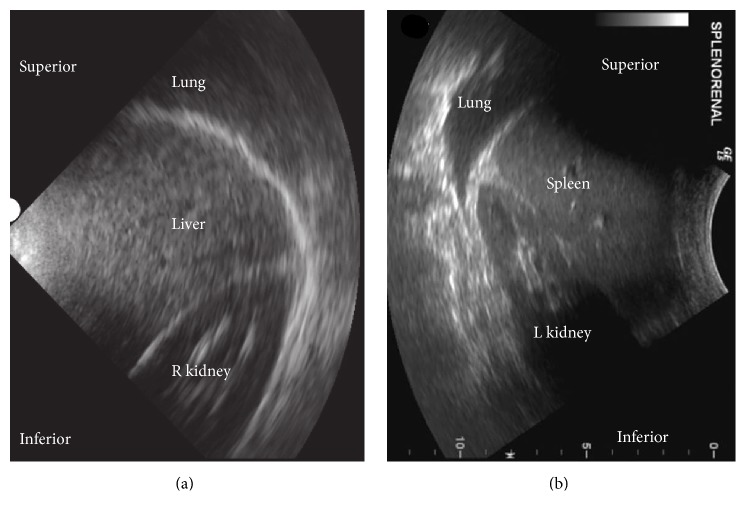
Renal assessment: ultrasound landmarks for kidneys. (a) RUQ hepatorenal view: landmarks for locating the kidney in the lateral right upper quadrant using a phased-array probe. The echogenic line separating the lung from the liver is the diaphragm (see Figure 4, probe position B). Adapted from Perera et al. with the authors' permission [27]. (b) LUQ splenorenal view: landmarks for locating the kidney in the lateral left upper quadrant using a curvilinear probe. The echogenic line separating the lung from the spleen is the diaphragm (see Figure 4, probe position C). Adapted from Montoya et al. with the authors' permission [1].

The kidneys can be visualized using either an abdominal or cardiac probe through the lower lateral ribs. The probe is held with the “knuckles to the bed” in the midaxillary line (Figures 4 and 6). Each kidney should be viewed in both the longitudinal and transverse planes, fanning through the entire kidney to view the cortex, medulla, and urinary pelvis. The maximum kidney length should be measured in the longitudinal view. Normal kidney length is approximately 10–12 cm with the left kidney longer than the right one by 0.3 cm [80]. Women, shorter people, and the elderly may have smaller kidneys. The cortex should be assessed for thickness and scaring. The renal cortex width is normally about 1 cm and the entire parenchyma is 1.5 cm [80]. The urinary pelvis should be evaluated for evidence of hydronephrosis which appears as branching, interconnected areas of decreased echogenicity that shows sonographic evidence of fluid. If the kidneys are difficult to locate, as in obesity, viewing from the costophrenic angle may be productive.

### 4.2. Clinical Utility

Bedside renal ultrasonography may be useful when the etiology of AKI is unclear, when the clinical course of AKI is not as expected or to differentiate AKI from AKI on CKD [10, 80]. Pertinent findings may include an estimate of kidney size and cortical thickness, moderate to large hydronephrosis, cysts, masses, or stones.

Small kidneys (short length) indicate CKD, while normal or increased kidney length may occur either in AKI due to ATN or nephritis or in CKD due to diabetes mellitus or other infiltrative causes [10]. Thinning of the renal parenchyma or cortex indicates CKD while normal cortical thickness may occur with CKD or AKI. Large cystic kidneys with cysts in the liver are consistent with autosomal dominant polycystic kidney disease. Small cystic kidneys indicate CKD. Kidney echogenicity is normally less than that of the liver or spleen, but increased echogenicity is not useful in distinguishing among the different causes of AKI, between AKI and CKD [80], or among interstitial fibrosis, tubular atrophy, inflammation, and glomerulonephritis [10].

Chronic partial urinary tract obstruction is usually associated with hydronephrosis. Obstruction without hydronephrosis can occur with retroperitoneal fibrosis or tumors, ureteral edema, or scarring as with tuberculosis or with early obstruction with inadequate time for dilation to occur. Hydronephrosis without obstruction can occur in pregnancy, vesicoureteral reflux, and megacystis-megaureter syndrome or after relief of obstruction; differentiation from obstruction requires further testing.

### 4.3. Comparison to the “Gold Standard”: Formal Renal Ultrasound

Bedside ultrasound is rapid and easy to perform, and findings are immediately available, while formal ultrasound has to be requested, performed, and interpreted, which results in some delay. Findings on bedside ultrasound can be verified by formal renal ultrasound at a later time, and additional testing can be requested as indicated. Hydronephrosis due to obstruction requires immediate intervention which may be delayed while waiting for formal ultrasound results.

### 4.4. Limitations

The kidneys may be hard to visualize, and the size may be underestimated if the longest dimension is not measured accurately [10]. False positives for hydronephrosis can occur with prominent hypoechoic pyramids or multiple renal cysts.

## 5. Summary

Over the past four years, we have used ultrasonography in intensive care units, the emergency department, wards, and clinics to visualize internal structures noninvasively and to assess physiologic function in real-time.

We qualitatively assess cardiac contractility and may find incidental pericardial effusions not detected by auscultation or physical examination. We use ultrasound of the inferior vena cava to estimate intravascular volume status and guide volume management. This is particularly crucial in hospitalized patients, who are not in steady state and frequently have mismatch: between intravascular volume and blood pressure or between intravascular and extravascular volume, which may not otherwise be evident on physical examination.

When we screen with bladder ultrasound, we frequently find incidental urinary retention or obstruction in patients with acute kidney injury, chronic kidney disease, or end-stage renal disease. Kidney ultrasound may provide an estimate of kidney size and disease chronicity or show moderate to large hydronephrosis or multiple cysts.

Further studies are needed to evaluate the benefit of nephrologists implementing a focused ultrasound protocol to improve the quality of the renal consultation.

## Figures and Tables

**Table 1:** Ultrasound findings in classic shock states.

Shock	Hypovolemic	Distributive	Cardiogenic	Obstructive
Heart	Hypercontractile Small chamber size	Hypercontractile (early sepsis) Hypocontractile (late sepsis)	Hypocontractile Dilated heart	Hypercontractile
				Pericardial effusion
				Cardiac tamponade
				RV strain
				Cardiac thrombus
IVC	Flat IVC	Normal or small IVC (early sepsis)	Distended IVC	Distended IVC

RV = right ventricle, IVC = inferior vena cava.

Adapted from Perera et al. with the authors' permission [4].

**Table 2:** Conditions biasing inferior vena cava ultrasound findings.

	IVC CI	IVCmax	Comments
*Underestimate intravascular volume*			
Increased tidal volume (ventilated)	Increased?	No change?	
Increased inspiratory effort moving probe “in & out” of field (diaphragmatic breathing) [18]	Increased	No change	Midaxillary or midclavicular line views [23]. Cross-sectional view [18]
Increased inspiratory effort/deep breathing (sniff) [22, 28, 35]	Increased	No change	Large IVCmax with no collapse indicates being not hypovolemic
Valsalva maneuver [19]	Increased	Decreased	Large IVCmax with no collapse indicates being not hypovolemic
Intra-abdominal HTN [23, 36]	?	Decreased	Large IVCmax with no collapse indicates being not hypovolemic.
Off-center scan (cylinder tangent effect) [37]	Minimal changes	Decreased	Attempt to maximize IVC diameter. Cross-sectional view [18]
*Overestimate intravascular volume*			
Cardiac tamponade	Decreased	Increased	Preload dependent
Severe valvular stenosis	Decreased	Increased	Preload dependent
Massive pulmonary embolism [18]	Decreased?	Increased	Preload dependent
Right ventricular myocardial infarction [38]	Decreased	Increased	Preload dependent, decreased venous return to LV
Severe tricuspid regurgitation	Decreased	Increased	
High PEEP [39]	Minimal change	Increased	No difference between PEEP 0 and 5 cm H_2_0[39]
Decreased tidal volume	Decreased	No change?	
Decreased inspiratory effort/shallow breathing [22, 40]	Decreased	No change?	Highly collapsible IVC indicates being not hypervolemic

IVC = inferior vena cava, IVC CI = IVC collapsibility index, IVCmax = IVC maximum diameter, PEEP = positive end-expiratory pressure, LV = left ventricle, and HTN = hypertension, cm H_2_0: centimeters of water.

**Table 3:** Mismatch between intravascular volume and blood pressure or extravascular volume.

*Mismatch between intravascular volume and blood pressure *	
States in which blood pressure is not primarily determined by intravascular volume	

Intravascular volume low Blood pressure high	Vasoconstriction
	(i) Stimulants (cocaine, amphetamines), catecholamines (pheochromocytoma, severe stress, delirium tremens)
	(ii) Severe hypothyroidism

Intravascular volume high Blood pressure low	Cardiac dysfunction
	(i) Cardiogenic shock
	(ii) Severe cardiomyopathy, heart failure, valvular heart disease
	Vasodilation
	(i) Distributive shock + excess volume resuscitation
	(ii) Autonomic neuropathy

*Mismatch between intravascular and extravascular volume*	

Intravascular volume low Extravascular volume high	Vasodilation and/or “third spacing”
	(i) Distributive shock (sepsis, anaphylaxis)
	(ii) Hemorrhagic pancreatitis
	(iii) Crush injury
	Delayed reequilibration
	(i) Severe renal failure + diuresis or ultrafiltration
	(ii) Nephrotic syndrome + diuresis
	(iii) End-stage liver disease + diuresis or large volume paracentesis or ultrafiltration
	(iv) Heart failure + diuresis or ultrafiltration

Intravascular volume high, Extravascular volume not high	Delayed reequilibration
	(i) Rapid blood transfusion + anuria or severe renal failure
	(ii) Rapid hypertonic sodium bicarbonate or saline infusion

**Table 4:** Comparison of techniques to assess intravascular volume and predict response to volume administration or removal.

Method	Circuit	Intervention	Threshold	“Gold standard”	SN (%)^*∗*^	SP (%)^*∗*^	Advantages	Disadvantages
Mean CVP [41] (*N* = 7)	Venous “static”	Central line Invasive	Mean CVP <8 mmHg	Increase of cardiac index ≥10–15%^*∗∗*^	76	62	None	Poor predictor of volume responsiveness [42]

Mean PAOP [43]	Pulmonary artery “static”	Central line Invasive	Mean PAOP <11 mmHg	Increase of cardiac index ≥15%^*∗∗*^	77	51	None	Poor predictor of volume responsiveness [42]

Right atrial pressure [44]	Venous “dynamic”	Central line Invasive	RAP variation ≥1 mmHg	Increase in CO >250 mL/min^*∗∗*^	91^@^	92^@^	“Dynamic” RAP predicts volume responsiveness	Not routinely available

IVC CI [41]	Venous “dynamic”	Ultrasonography Noninvasive		Increase of cardiac index ≥10–15%^*∗∗*^			Can differentiate overt volume depletion from overt overload. Ventilated or nonventilated patients.Readily available	Requires further validation. Operator dependent. Requires practice. Intermittent monitoring
Ventilated (*N* = 4)Tidal volume >8 mL/kg			IVC DI >15% = IVC CI >9.4%		77	85		
Spontaneous breathing (*N* = 2)			IVC CI >41%^#^		31, 70	97, 80		

IVC CI [45]^$^	Venous “dynamic”	Ultrasonography Noninvasive	IVC CI <20%	Removal of >1 L by UF	64	64	As above	As above

IVCmax + IVC CI [46]	Venous “dynamic”	Ultrasonography Noninvasive	IVCmax >2cm + IVC CI <50%	Mean RAP >10 mmHg	82	67	As above	As above

RUSH exam [5]^$^	Heart/venous “dynamic”	Ultrasonography Noninvasive	Cardiac function IVCmax + IVC CI	Final diagnosis of type of shock	88	96	As above	As above

Arterial line wave form analysis [41]	Arterial “dynamic”	Arterial line with standard multiparameter monitor Invasive		Increase of cardiac index ≥10–15%^*∗∗*^			Minimally invasive. Continuous monitoring	Only validated with mechanical ventilation, no spontaneous breaths, and no arrhythmias. Less reliable in automated systems [47]. Not validated in hypervolemia [48, 49]
Tidal volume <7 mL/kg (*N* = 5)			PPV ≥8%		72	91		
Tidal volume ≥7 mL/kg (*N* = 17)					84	84		
Controlled ventilation (*N* = 9)			SVV ≥13%		82 [48]	84–86 [41, 48]		

Bioreactance + passive leg raising [50]^$^	Arterial “dynamic”	NICOM/ Cheetah^&^apparatus Noninvasive	Increase of SVI >10% after PLR	Increase of SVI >10% after volume administration	94	100	Continuous monitoring. Ventilated or nonventilated patients	Not validated in hypervolemia. Equipment may not be readily available

^*∗*^Sensitivity and specificity to predict response to volume administration or removal. Summary values for data from meta-analysis from Bentzer et al. [41] unless otherwise referenced.

^*∗∗*^After volume administration.

^@^Only 13 of 14 data points for nonresponders and 17 of 19 data points for responders were extractable from the figure.

^#^No sniff or valsalva.

^$^Heterogeneous population with ventilated and nonventilated, pressors or no pressors, multiple comorbidities.

^&^Cheetah Medical Inc., Portland, OR, USA.

SN = sensitivity, SP = specificity, CVP = central venous pressure, *N* = number of studies from Bentzer et al. [41], PAOP = pulmonary artery occlusion pressure, CO = cardiac output, RAP = right atrial pressure, IVC CI = inferior vena cava collapsibility index, IVC DI = inferior vena cava distensibility index, UF = ultrafiltration, IVCmax = inferior vena cava maximum diameter, RUSH = rapid ultrasound in shock, PPV = pulse pressure variation, SVV = stroke volume variation, NICOM = noninvasive cardiac output monitor, SVI = stroke volume index, and PLR = passive leg raising.
